# Synergistic role of MoS_2_ in gelation-induced fabrication of graphene oxide films

**DOI:** 10.1038/s41598-024-62146-4

**Published:** 2024-05-28

**Authors:** Minah Choi, Joonwon Lim, Jieun Yang

**Affiliations:** 1 Department of Chemistry, College of Science, Kyung Hee University, 26 Kyungheedae-ro, Dongdaemun-gu, Seoul, 02447 Republic of Korea; 2 Department of Information Display, College of Science, Kyung Hee University, 26 Kyungheedae-ro, Dongdaemun-gu, Seoul, 02447 Republic of Korea

**Keywords:** Materials science, Nanoscience and technology

## Abstract

Supporting materials for electrocatalysts must exhibit relative chemical inertness to facilitate unimpeded movement of gas, and demonstrate electrical conductivity to promote efficient electron transfer to the catalyst. Conventional catalyst electrodes, such as glassy carbon, carbon cloths, or Ni foam, are commonly employed. However, the challenge lies in the limited stability observed during testing due to the relatively weak adhesion between the catalyst and the electrode. Addressing this limitation is crucial for advancing the stability and performance of catalyst-electrode systems in various applications. Here, we suggest a novel fabrication method for a freestanding conducting film, accomplished through gelation, incorporating 1T-MoS_2_ and graphene oxide. 1T-MoS_2_ nanosheets play a crucial role in promoting the reduction of graphene oxide (GO) on the Zn foil. This contribution leads to accelerated film formation and enhanced electrical conductivity in the film. The synergistic effect also enhances the film’s stability as catalyst supports. This study provides insights into the effective utilization of MoS_2_ and graphene oxide in the creating of advanced catalyst support systems with potential applications in diverse catalytic reaction.

## Introduction

In electrochemical reaction, supporting materials are important in defining electrocatalyst properties such as durability, selectivity and activity^1,2^. Electrocatalytic materials, including metal nanoparticles, single atom catalysts, and metal oxide particles are capable of being dispersed and anchored onto a supporting material. The ideal supporting materials should possess a substantial surface area, be relatively chemically inactive to promote a passage for gas or liquid molecules freely to move and possess electrical conductivity to facilitate electron transfer to the catalysts. In addition, the supporting material should be able to endure long cycles and remain stable in acidic or alkaline electrolytes. Carbon black is a widely favored option due to its porous structure, conductivity, and low cost. However, it requires thermal annealing before use and has limited electrochemical stability. Hence, researchers are exploring alternative support materials to overcome these drawbacks^3^.

Layered materials like graphene, reduced graphene oxide (rGO), and transition metal dichalcogenides (TMDs) often used as support materials for electrocatalysts. rGO nanosheets are particularly popular due to their high electrical conductivity, large surface area, and mechanical strength. Meanwhile, molybdenum disulfide (2H-MoS_2_), a type of TMDs, has an analogous structure to rGO, thickness -dependent band gap and phase-dependent electrical properties. The large interlayer spacing of 2H-MoS_2_ allows for the insertion of alkali metals between the layers of MoS_2_, which results in loss of semiconducting properties due to the emergence of a metallic 1T phase. Nevertheless, 1T-MoS_2_ nanosheets and rGO both have high electrical conductivity; the electrical conductivity of rGO changes depending on the reduction method, the electrical conductivity of thermally annealed rGO under CH_4_/H_2_, C_2_H_2_, and CH_4_ plasma is around 350–410 S/cm, 1425 S/cm, and 1590 S/cm, respectively^4,5^. Chemically reduced GO shows around 100–1000 S/cm^6^. While 1T-MoS_2_ possesses a conductivity of 400–600 S/cm, fabricating large-are free-standing thin films is challenging due to its weak mechanical strength^7^. Consequently, rGO/MoS_2_ thin films hold promise as they can potentially overcome the limitations associated with individual components, providing a synergistic combination of favorable properties.

The combination of two individual components in the rGO hybrid structure has resulted in a synergistic effect that has been successfully applied to various fields. These hybrid nanosheets have proven to be better than single products for a variety of applications including photocatalysis, batteries, and energy harvesting showed better performance^8,9^. This enhanced performance of MoS_2_/rGO films is mainly attributed to the synergistic effect between several layers of MoS_2_ and rGO sheets. Therefore, several attempts have been made including simple mixing and vacuum filtration, as well as hydrothermal methods^8,10^. While the simple mixing can lead to large production, it is a time-consuming process with size limitations and random stacking. The hydrothermal methods allow for a more uniform nanoscale structure, but the morphologies are not ideal for electrocatalysts support material. Additionally, most techniques produce semiconducting 2H–MoS_2_ instead of metallic 1T-MoS_2_, which is desired because the 1T phase returns to the 2H phase at temperatures over 200°C^11^. To circumvent the loss of the metallic nature of the 1T phase, it is recommended to avoid the high temperature.

As an alternative approach, gelation can be employed to prepare supporting materials utilizing rGO/MoS_2_ nanosheets, which is a simple and easy process. Gelation offers an advantageous for large-scale production without size limitation^12,13^. Consequently, we fabricated large-area catalyst supports via gelation using GO and 1T-MoS_2_. This process does not require a further reduction treatment, there by enabling the retention of the metallic properties of 1T-MoS_2_. Remarkably, the addition of 1T-MoS_2_ was observed to facilitate a rapid reduction of GO to rGO. The rGO/MoS_2_ film obtained via gelation exhibited stable in acidic and alkaline electrolyte solutions and could be utilized as a catalyst support for water-splitting reactions. Furthermore, it was possible to obtain large-area free-standing films, with the size dependent on the dimensions of the Zn foil employed.

## Methods

### Preparation of graphene oxide (GO)

GO was prepared by the improved Hummer’s method^14^. 0.5 g of NaNO_3_ (99.0%, Alfar Aesar) and 1.0 g of graphite flakes was mixed with 50 mL of H_2_SO_4_ (95.0–98%, Sigma-Aldrich) in an ice bath. 3.0 g of KMnO_4_ (≥ 99%, Sigma-Aldrich) was added slowly in the bath. The mixture was stirred for 5 h at 35 °C. An additional 3.0 g of KMnO_4_ was added, and the reaction was stirred for a further 12 h at the same temperature. The resulting mixture was cooled to room temperature and quenched by pouring it onto ice along with 30% H_2_O_2_. The solution was thoroughly washed with 100 mL of distilled water and 100 mL of HCl, with each washing step being followed by centrifugation at 3000 rpm for 30 min. The final product was dialyzed over a period of 2–3 days.

### Exfoliation of MoS_2_

0.3 g of molybdenum sulfide(MoS_2_, metal basis, 99%, Alfa Aesar) and 20 mL of hexane (mixture of isomers, anhydrous, ≥ 99%, Sigma-Aldrich) were purged with argon for 10 min. Subsequently, 3 mL of n-butyllithium solution (1.6 M in hexanes, Sigma-Aldrich) was added, and the temperature was gradually increased to 90 °C under Ar purging conditions for 30 min. The mixture was refluxed for 48 h. Li-intercalated MoS_2_ was washed with 150 mL of hexane, then dispersed to 0.1 mg mL^−1^ with distilled water. A bath sonicator (Elmasonic P30H ultrasonic cleaner) was used for sonicating (80 kHz frequency, 100% power) dispersed MoS_2_ for 1 h. After sonication, MoS_2_ solution was centrifuged at 10,000 rpm twice for 1 h and removed supernatant. Finally, the centrifugation was proceeded again to collect supernatant at 3000 rpm twice for 1 h.

### Gelation of MoS_2_/rGO nanosheets

GO synthesized by Hummer’s method was diluted with distilled water and subsequently mixed with a 15-fold diluted 0.1 M HCl solution (36%, Alfar Aesar), followed by the addition of 1T-MoS_2_. The Zn foil(thickness 0.25 mm, 99.8%, Sigma-Aldrich) was then immersed in the acidic MoS_2_/GO solution^12^. Within 5 min, MoS_2_/rGO hydrogel formed on the surface of the Zn foil. The sample was then washed with distilled water to remove physically adsorbed GO. Subsequently, an etching process was carried out using 0.5 M HCl. Finally, free-standing MoS_2_/rGO nanosheets were obtained by rinsing with distilled water to eliminate acidic impurities.

### Vacuum filtration of rGO/1T-MoS_2_

A 50 mL of GO solution with a concentration of 1.0 mg/mL was initially prepared. 2 μL of hydrazine was added to the solution, followed by stirring at 80 °C for 1 h to facilitate reduction. Afterward, the reduced graphene oxide (rGO) solution was subjected to bath sonication to ensure thorough mixing with a 1T-MoS_2_ solution. Next, the solution was filtered to form a film, followed by drying to remove solvent in ambient.

### Electrochemical measurements

Electrochemical measurements were performed using a VSP potentiostat from BioLogic. An Ag/AgCl electrode (3 M NaCl, BAS Inc.) and graphite rod were used as the reference electrode and counter electrode, respectively. Measurement of reduction potential were executed using glassy carbon electrode (diameter = 3 mm) as the working electrode coated with 10 μL of GO, 1T-MoS_2_ and 1T-MoS_2_/GO solutions. Cyclic voltammetry was performed in 0.0001 M H_2_SO_4_ at a scan rate of 5 mV s^−1^. Hydrogen evolution reaction (HER) activities and stability tests were carried out in 0.5 M H_2_SO_4_. The working electrode for this purpose was the 1T-MoS_2_/rGO film, fabricated via the gelation method, securely affixed to a glass slide using silver/epoxy. Platinum on carbon (5 wt%, Sigma-Aldrich) was dispersed into 1 mg mL^−1^ with distilled water and coated onto the 1T-MoS_2_/rGO film. Linear sweep voltammetry was performed at a scan rate of 5 mV s^−1^. Chronoamperometry were measured at − 0.5 V versus a reversible hydrogen electrode (RHE) for 32 h. To test the inactivity of the film in the electrolyte, linear sweep voltammetry was performed in 0.5 M H_2_SO_4_ and 1 M KOH. The working electrode in these experiments consisted of a glassy carbon electrode coated with the 1T-MoS_2_/rGO hydrogel. For measurements in an alkaline electrolyte, a calomel electrode (1 M NaOH, BAS Inc.) was used as the reference electrode. The potentials were referenced to RHE, calculated as E(V vs. RHE) = E(Ag/AgCl) + 0.209 V + 0.059 $\times$ pH in acidic conditions and E(V vs. RHE) = E(Calomel) + 0.140 V + 0.059 $\times$ pH in alkaline conditions.

## Results and discussion

**Figure 1:**
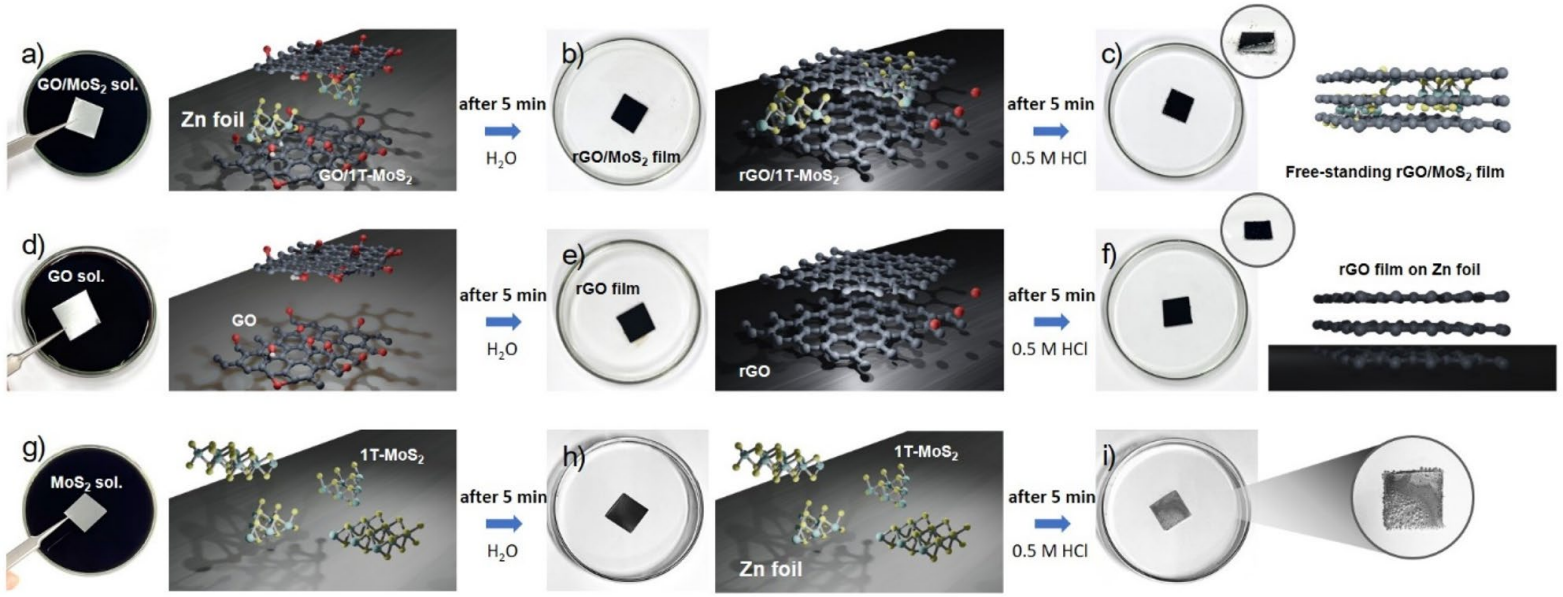
Photographs and schematic illustrations of gelation method. (**a, b, c**) Fabrication of rGO/1T-MoS_2_ film. (**a**) Zn foil is immersed in an acidic GO/1T-MoS_2_ solution. (**b**) The rGO/1T-MoS_2_ hydrogel is stacked on the Zn foil after a 5-min immersion time. (**c**) The detachment of the hydrogel films formed on the Zn foil is carried out through an etching process in 0.5 M HCl within 5 min. The gelation was also performed in (**d, e, f**) GO and (**g, h, i**) 1T-MoS_2_ solutions following a same procedure as previously described. (**d, e**) In the GO solution, the reduction of GO and the formation of hydrogel occur simultaneously similar to what was observed in (**b**) GO/MoS_2_; nevertheless, (**f**) the separation of the rGO film takes more than 1 h. (**g, h**) In the case of 1T-MoS_2_, the hydrogel did not stacked on the Zn foil even after 24 h.

**Figure 2:**
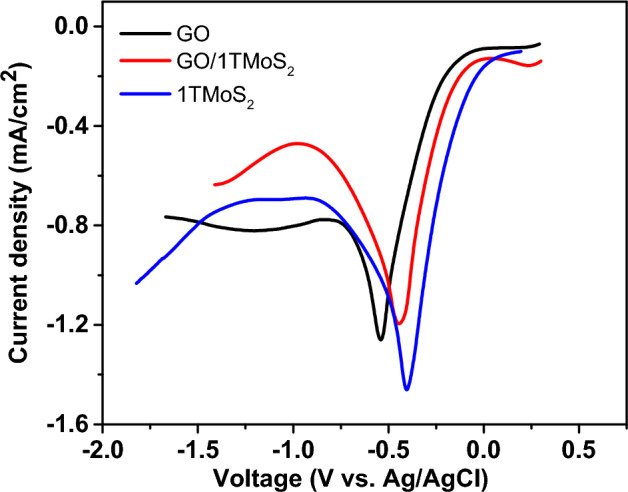
Linear sweep voltammograms of GO, GO/1T-MoS_2_, and 1T-MoS_2_.

The gelation method is known for preparing a freestanding rGO film using a reduction potential difference between a metal foil and GO. In this process, when a zinc foil is immersed in a GO dispersion solution, GO accumulates on the foil’s surface while concurrently undergoing reduction, thus leading to the formation of an rGO film. The reduction potential of rGO/GO (− 0.4 V vs. SHE at pH 4) is higher than that of Zn/Zn^2+^ (− 0.7 V vs. SHE)^12^. Therefore, as the Zn surface is ionized under acidic conditions, reduction of GO can occur spontaneously. Here, we integrated metallic 1T-MoS_2_ sheets with GO sheets to obtain a film that exhibits higher conductivity in comparison to a rGO film. The synthetic procedure for rGO/1T-MoS_2_ film is shown in Fig. 1. To fabricate rGO/1T-MoS_2_ film, chemically exfoliated 1T-MoS_2_ sheets are introduced into the GO solution. Within a short duration of 5–10 min, the black rGO/1T-MoS_2_ sheets are assembled spontaneously and covered on the Zn surface in varying thicknesses contingent upon immersion time. The film formed on the Zn foil was subsequently separated from the Zn substrate through a gentle acid etching process, after which the detached film underwent freeze-drying. We systematically observed how films are formed when GO sheets and 1T-MoS_2_ sheets coexist, as well as when GO sheets and 1T-MoS_2_ are present, independently. In the presence of 1T-MoS_2_, a 10 μm-thick rGO/1T-MoS_2_ film manifested within 5 min, whereas the formation of GO film, in the absence of 1T-MoS_2_, necessitated approximately 30 min to achieve a comparable thickness of 10 μm. This discrepancy highlights the considerably shorter film formation time when GO sheets are combined with 1T-MoS_2_, as opposed to GO sheets alone. To further elucidate the relation behind this accelerated formation process, we conducted measurement of the electrochemical reduction potential of the GO/1T-MoS_2_ film in H_2_SO_4_ at pH 4, as presented in Fig. 2. Surprisingly, the reduction potential of the GO/1T-MoS_2_ increased up to − 0.44 V vs. Ag/AgCl. Indeed, the elevated reduction potential played a pivotal role in expediting the film formation process. Conversely, when considering 1T-MoS_2_ sheets in isolation, they do not exhibit stacking behavior on the Zn foil, as illustrated in Fig. 1h. Consequently, the interfacial gelation of GO/1T-MoS_2_ occurs in accordance with the following reaction, particularly under acidic condition:

$${\text{GO}}/{\text{1T}} - {\text{MoS}}_{{2}} + {\text{ Zn }} + {\text{ H}}^{ + } \to {\text{rGO}}/{\text{1T}} - {\text{MoS}}_{{2}} + {\text{ Zn}}^{{{2} + }} + {\text{ H}}_{{2}} {\text{O}}$$

**Figure 3:**
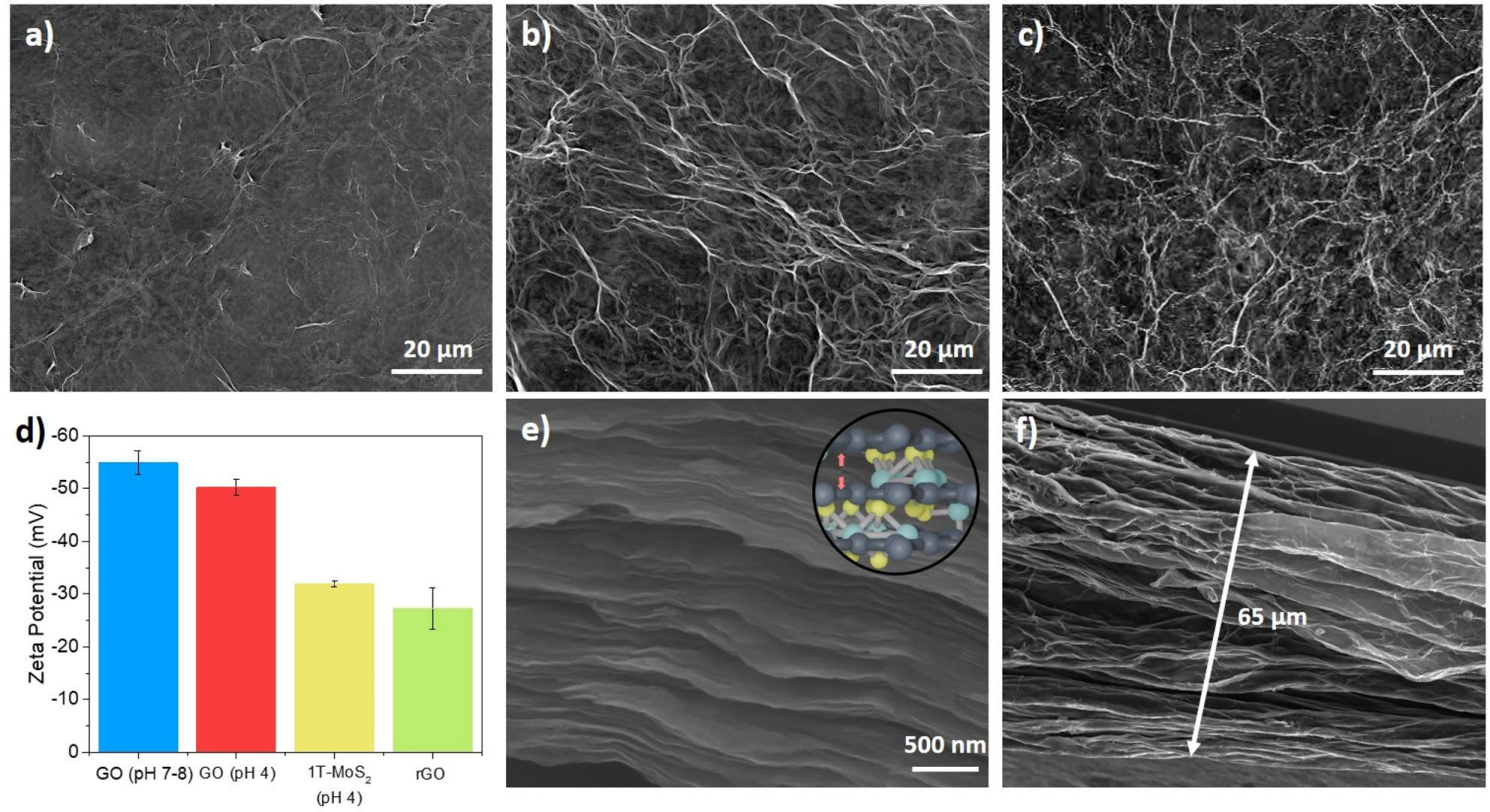
SEM images of GO (**a**), rGO/1T-MoS_2_ ((**b**), with a volume ratio of rGO to MoS_2_ = 7:1), and rGO/1T-MoS_2_ ((**c**), with a volume ratio = 7:5). (**d**) Zeta potential of GO and 1T-MoS_2_ according to pH. (**e and f**) Cross-sectional images of rGO/1T-MoS_2_ film.

The surface morphology of the film was examined using scanning electron microscope (SEM) (Fig. 3). An increase in the concentration of 1T-MoS_2_ led to the emergence of noticeable surface wrinkles. This phenomenon is ascribed to the electrostatic interactions that develop between GO and 1T-MoS_2_. Specifically, the presence of oxygen functional groups, such as hydroxyl groups, on the surface of GO results in a negative charge (zeta potential: − 55 mV). Even when GO is exposed to acidic conditions (0.1 M HCl), the zeta potential does not exhibit a significant difference. The dispersion of 1T-MoS_2_ carries a less negative charge compared to GO, inducing attractive forces between GO and 1T-MoS_2_ which can be attributed to the electrostatic interactions that develop between rGO and 1T-MoS_2_. Intriguingly, owing to the relatively small quantity of 1T-MoS_2_ added compared to GO, the stacking of sheets on the Zn foil takes place gradually rather than instantaneously aggregating.

**Figure 4:**
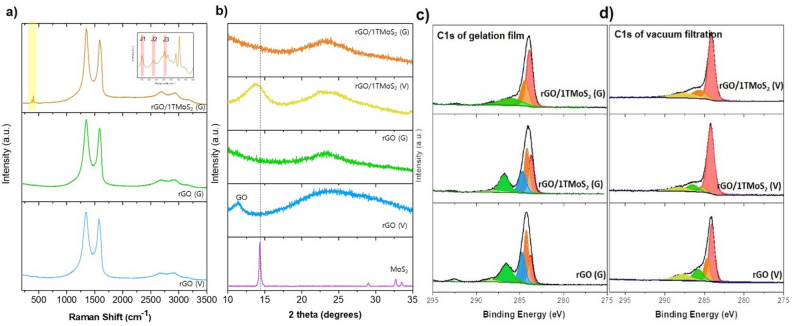
(**a**) Raman spectra of rGO via vacuum filtration film (rGO(V)), rGO(G) and rGO/1T-MoS_2_(G) film made of gelation. (**b**) XRD spectra of MoS_2_, rGO(V), rGO(G), rGO/1T-MoS_2_(V), and rGO/1T-MoS_2_(G). The dash line indicates (200) of MoS_2_. (**c**) XPS C1s spectra of rGO(G) (bottom), rGO/1TMoS_2_(G) (middle) with a volume ratio rGO to 1T-MoS_2_ = 7:1, and rGO/1TMoS_2_(G) (top) with a volume ratio = 7:5. (**d**) XPS C1s spectra of vacuum filtration film. rGO/1TMoS_2_(V) (middle) with a volume ratio rGO to 1T-MoS_2_ = 7:1, and rGO/1TMoS_2_(V) (top) with a volume ratio = 7:5. rGO is prepared by hydrazine reduction.

The structural characteristics of the rGO/1T-MoS_2_ film were comprehensively analyzed using Raman spectroscopy, X-ray photoelectron spectroscopy (XPS), and X-ray diffraction (XRD). The film generated through gelation is denoted as rGO/1T-MoS_2_(G). In the Raman spectrum (Fig. 4a), two prominent bands, G and D, were identified at 1593 cm^−1^ and 1344 cm^−1^, respectively, for GO. However, in the case of the rGO/1T-MoS_2_ film, these bands exhibited a slight downshift, appearing at 1585 cm^−1^ and 1343 cm^−1^, respectively. This downshift of the G band and the narrowing of the D and G bands collectively signify the reduction of GO within the film^12^. Additionally, the Raman spectrum of 1T-MoS_2_ revealed three distinctive peaks located at 283.5 cm^−1^, 381.2 cm^−1^ and 405.3 cm^−1^ corresponding to the E_1g_, E_2g_ and A_1g_ vibrational modes, respectively, which are indicative of the specific crystalline structure of 1T-MoS_2_. The J peaks related to 1T-phase character appear at 155.5 cm^−1^, 225.8 cm^−1^, and 320 cm^−1^ for J_1_, J_2_ and J_3_, respectively, which means that metallic property remains unchanged during the formation of the film^11^. The intensity of Raman spectrum of 1T-MoS_2_ (inset in Fig. 4a) appeared lower than that of GO because the used concentration of 1T-MoS_2_ is relatively low. In the Raman spectrum of rGO/1T-MoS_2_(G), it can be observed that MoS_2_ is uniformly distributed in the gelation film. It indicates the individual 1T-MoS_2_ sheets are embedded into rGO sheets homogeneously in rGO/1T-MoS_2_ (G). In contrast, MoS_2_ in the vacuum filtration (rGO/1T-MoS_2_(V)) film is randomly distributed, leading to infrequent observations of MoS_2_ flakes (Figure S1). Figure 4b displays the XRD spectra the vacuum-filtration film and the gelation film. In the rGO(V) film, the XRD peak at 11.4° indicates the presence of unreduced GO, while the broad peak around 23° signifies reduced GO(rGO). In rGO/1T-MoS_2_(V) prepared via vacuum filtration, the peak at 14.4° corresponding to the stacking of 1T-MoS_2_, as well as peak associated with rGO, were observed. In contrast, the peak at 14.4° in rGO/1T-MoS_2_(G) was not observed. This result strongly suggests that 1T-MoS_2_ sheets in the rGO/1T-MoS_2_ (G) were embedded into rGO by single layers because multilayer 1T-MoS_2_ sheets appear at the peak of 14.4. This observation is supported by the Raman spectrum, where in rGO/1T-MoS_2_(G), the consistent presence of 1T-MoS_2_ across different film positions was confirmed. Furthermore, elemental mapping confirmed the homogeneous distribution of MoS_2_ in rGO/1T-MoS_2_ (G) film (Figure S2). However, in rGO/1T-MoS_2_(V), the irregular appearance of the 1T-MoS_2_ peak depended on the film position. This irregularity arises through vacuum filtration, potentially leading to localized accumulation. Therefore, both Raman and XRD analyses indicate that in the film formed through gelation, MoS_2_ and rGO are uniformly distributed between them. The degree of reduction was observed by XPS. The estimated carbon/oxygen atomic ratio from Fig. 4c shows 0.8, 1.2, and 3.6 for rGO(G), rGO/1TMoS_2_(G) with a volume ratio rGO to 1T-MoS_2_ = 7:1, and rGO/1TMoS_2_(G) with a volume ratio = 7:5, respectively. As the amount of MoS_2_ increases, the number of oxygen functional groups decreases, showing that MoS_2_ is effective in reduction GO. The identical experiments were conducted using Fe foil to validate this phenomenon. (Figure S3) It is clearly evident that the formation of the film occurred more rapidly and efficiently in solutions contacting MoS_2_. The deconvoluted C1s XPS spectrum shows the two peaks for the sp^2^ (C = C) and sp^3^ (C–C) structure at 284.3 eV and 284.8 eV, respectively^15,16^. The intensity of sp^3^ peak and oxygen functional groups (C–O at 285.5 eV and O–C = O at 287.5 eV) of rGO/1T-MoS_2_ film has decreased compared to rGO, which means it is highly reduced during the gelation. For a precise comparison, we assessed it against a film produced through vacuum filtration (Fig. 4d). Notably, there is no substantial difference compared to the film reduced with hydrazine, suggesting the effective removal of oxygen functional groups.

**Figure 5:**
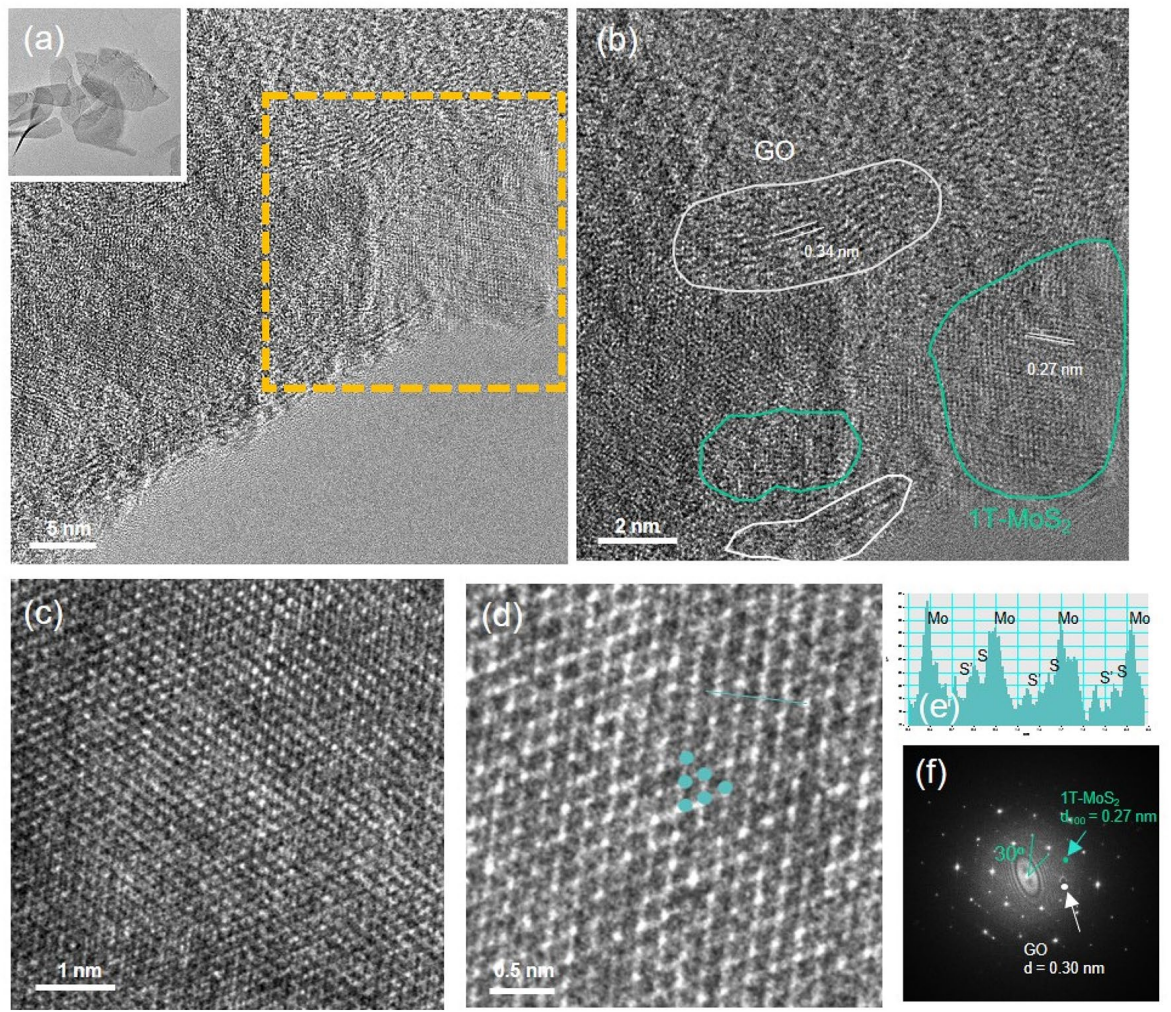
TEM images of the GO/1T-MoS_2_ composite. (**a**) GO/1T-MoS_2_ sheets. (**b**) Magnified view of the marked area in (**a**). As indicated, the white regions represent GO areas with a d-spacing value of 0.34 nm while the green regions suggest the presence of MoS_2_ domains corresponding to a d-spacing of 0.27 nm. (**c**) and (**d**) High-magnification views of the MoS_2_ regions, directly revealing the 1T phase of MoS_2_. (**e**) Atomic arrangement profile along the marked line in (**d**). (**f**) Fast Fourier transform (FFT) pattern of rGO/MoS_2_. The 30° angular spacing between the hexagonal spots assigned to MoS_2_ indicates the presence of 1T-MoS_2_ in rGO/MoS_2_ film.

To investigate the phase of MoS_2_ within the rGO/MoS_2_ composite even after the gelation process, transmission electron microscopy (TEM) analysis was performed. The TEM analysis confirmed the presence of MoS_2_ in the 1T phase. The TEM images revealed a uniform dispersion of graphene oxide and MoS_2_ within the composite (Fig. 5a and b). The magnified images and the corresponding atomic arrangement profiles directly demonstrated the 1T phase of MoS_2_ (Fig. 5c–f). Furthermore, the observed fast Fourier transform (FFT) pattern corroborated the homogeneous mixing of graphene oxide and MoS_2_ within the composite.

**Figure 6:**
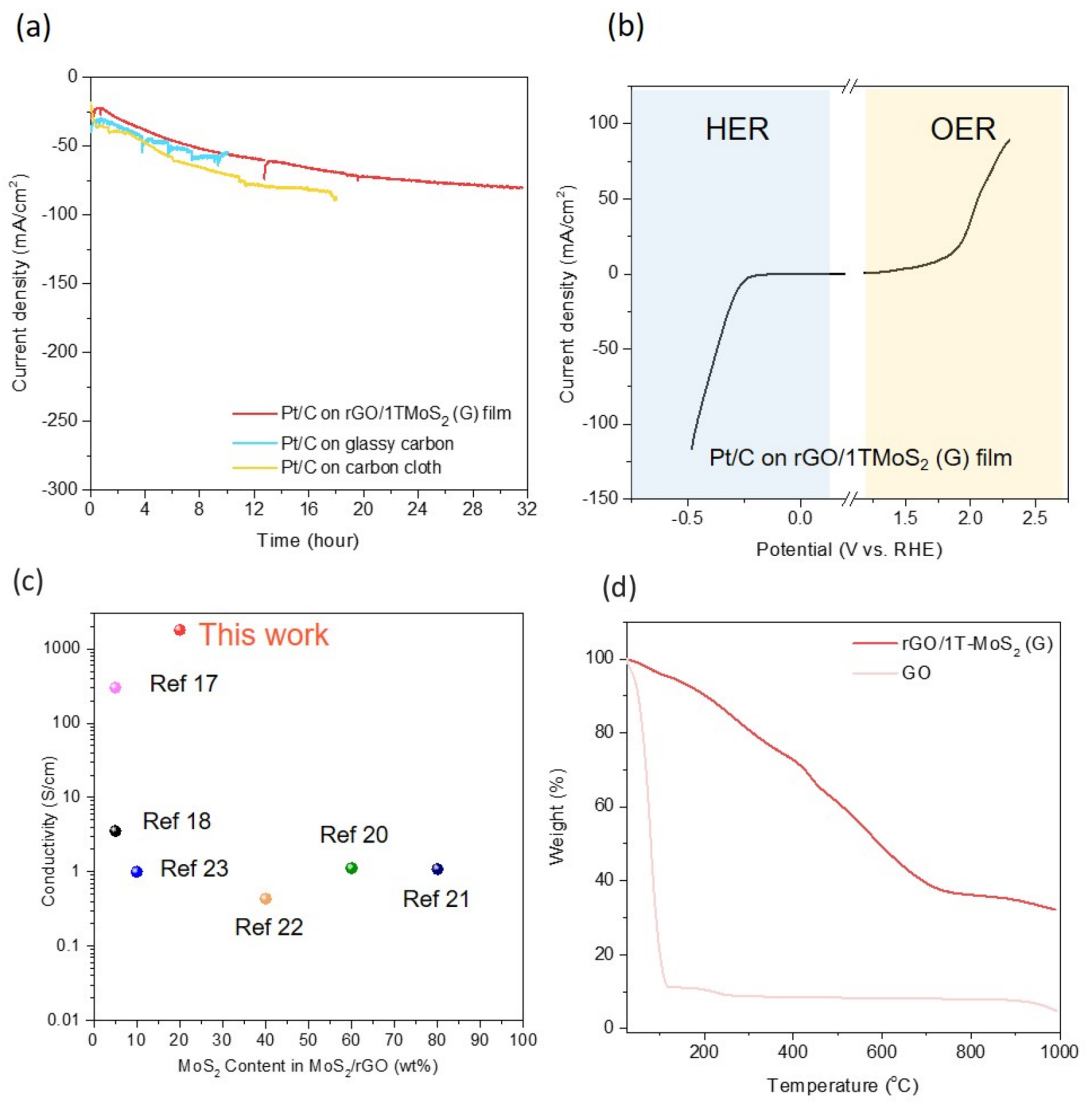
(**a**) Chronometric testing of Pt/C on glassy carbon, carbon cloths, and rGO/1TMoS_2_ (G) film at − 0.5 V vs RHE in acidic electrolyte. (**b**) Polarization curves for HER and OER in alkaline electrolyte. (**c**) Comparison of conductivity as a function of MoS_2_ content by weight (wt%). (**d**) MoS_2_ content is measured by thermogravimetric analysis (TGA).

The free-standing film produced through gelation is suitable as catalyst support because it consumes little time, has no size limitation. In order to test the rGO/1T-MoS_2_ film by gelation as supports, Pt/C catalysts were deposited on three different substrates—glassy carbon, carbon cloth, and rGO/1TMoS_2_ film. Chronoamperometric tests were conducted to assess the electrochemical stability of the deposited catalysts. Remarkably, the Pt/C on rGO/1T-MoS_2_(G) demonstrated stability for more than 32 h without the use of Nafion and binders (Fig. 6a).

To investigate the intrinsic catalytic activity of the catalyst, the use of binders was avoided, and the encouraging result indicates that the rGO/1T-MoS_2_(G) film remains stable for several days without a binder. The hydrophilic nature of the rGO/1T-MoS_2_(G) film, in comparison to glassy carbon or carbon cloth, facilitates excellent contact with the catalysts. Electrochemical reactions usually suffer from weak adhesion between the catalysts and the electrodes. In fact, the catalyst coated on glassy carbon tended to detach within 2–3 h due to the increased generation of hydrogen and oxygen bubbles during electrochemical reactions. Therefore, instead of glassy carbon, Ni foam or carbon cloth is employed as a support. Ni foam, being a metal, can actively participate in the catalytic reaction, while the surface of carbon cloth, being hydrophobic, requires an additional process to enhance its hydrophilicity for improved contact with the catalyst. Not only did the rGO/1T MoS_2_ (G) film exhibit superior stability, but it was also observed to enable electrochemical reaction measurements over a wide potential range (Fig. 6b). As supporting materials, rGO/MoS_2_ film doesn’t show the catalytic activities for HER and OER and electrochemical surface area (ECSA) was measured. (Figure S4 and S5) The electrical conductivity of the majority of rGO/MoS_2_ composites typically decreases with an increase in MoS_2_ content^8–10,17–20^. However, in the case of the rGO/1T-MoS_2_(G) film, the electrical conductivity slightly increased compared to the rGO film. The sheet resistance (R_s_) of rGO/1T-MoS_2_ (G) film is 50–100 Ω/sq which gives a high conductivity (σ) around 400–500 S/cm (calculated with $\upsigma =1/{R}_{s}\times$ t, where t is the film thickness, about 0.5 μm, Fig. 6c). This can be attributed to the even distribution of metallic 1T-MoS_2_ sheets throughout the rGO matrix. Therefore, 1T-MoS_2_/rGO film shows higher conductivity and low sheet resistance which are suitable for catalyst support. (Table S1) The MoS_2_ content in rGO/MoS_2_ (G) film is measured by TGA (Fig. 6d). For the gelated GO, weight loss was observed near 150 °C and 220 °C attributable to the desorption of water molecules and the breakdown of oxygen functional groups, respectively. The significant weight loss between 100 and 300 °C indicates that the gelated GO was partially reduced^21^. In comparison to the gelated GO, the rGO/MoS_2_ exhibited improved thermal stability below 600 °C suggesting effective reduction of GO in the presence of MoS_2_. Three distinct weight loss regions were observed for rGO/MoS_2_. The weight loss near 150 °C corresponds to the elimination of adsorbed water species. The weight loss around 400 °C indicates the decomposition of residual oxygen functional groups. Complete decomposition occurred at 700 °C. The observed 70% weight loss implies that 30% of the composite consists of MoS_2_. MoS_2_ does not undergo significant weight loss as it oxidizes to MoO_3_^9^.

## Conclusion

We have developed an efficient method for fabricating a catalyst support. Notably, GO was successfully reduced without resorting to time-consuming procedures such as thermal annealing or chemical treatment. Moreover, the inclusion of metallic 1T-MoS_2_ led to a notable enhancement in the electrical conductivity of the resulting film. The fabricated film exhibits several desirable characteristics: it’s stable in both alkaline and acidic electrolytes, and demonstrates excellent contact between catalysts and the film substrates. Although the electrical conductivity of the rGO/1T-MoS_2_ film is lower compared to that of commercial glassy carbon, its suitability for applications requiring large-area catalyst supports remains evident. In summary, our approach offers a promising method for the scalable fabrication of catalyst supports, leveraging the synergistic properties of 1T-MoS_2_ and rGO, and showing favorable characteristics for practical applications.

### Supplementary Information

Supplementary Information.

## Data Availability

All data generated during this study are included in this article.
