# Supplementary material for: Synergistic role of MoS2 in gelation-induced fabrication of graphene oxide films
Source: Sci Rep. 2024 May 28;14:12159. doi: 10.1038/s41598-024-62146-4 (PMC11130228; doi:10.1038/s41598-024-62146-4)
Supplement: Supplementary file 1 — Supplementary Information. [file 41598_2024_62146_MOESM1_ESM.docx]

Supporting Information

**Synergistic Role of MoS_2_ in Gelation-Induced Fabrication of Graphene Oxide films**

Minah Choi^1^, Joonwon Lim^2*^, and Jieun Yang,^1*^

^1^Department of Chemistry, College of Science, Kyung Hee University, 26 Kyungheedae-ro, Dongdaemun-gu, Seoul 02447, Republic of Korea

^2^ Department of Information Display, College of Science, Kyung Hee University, 26 Kyungheedae-ro, Dongdaemun-gu, Seoul 02447, Republic of Korea

E-mail: joonwon.lim@khu.ac.kr, jey@khu.ac.kr

.


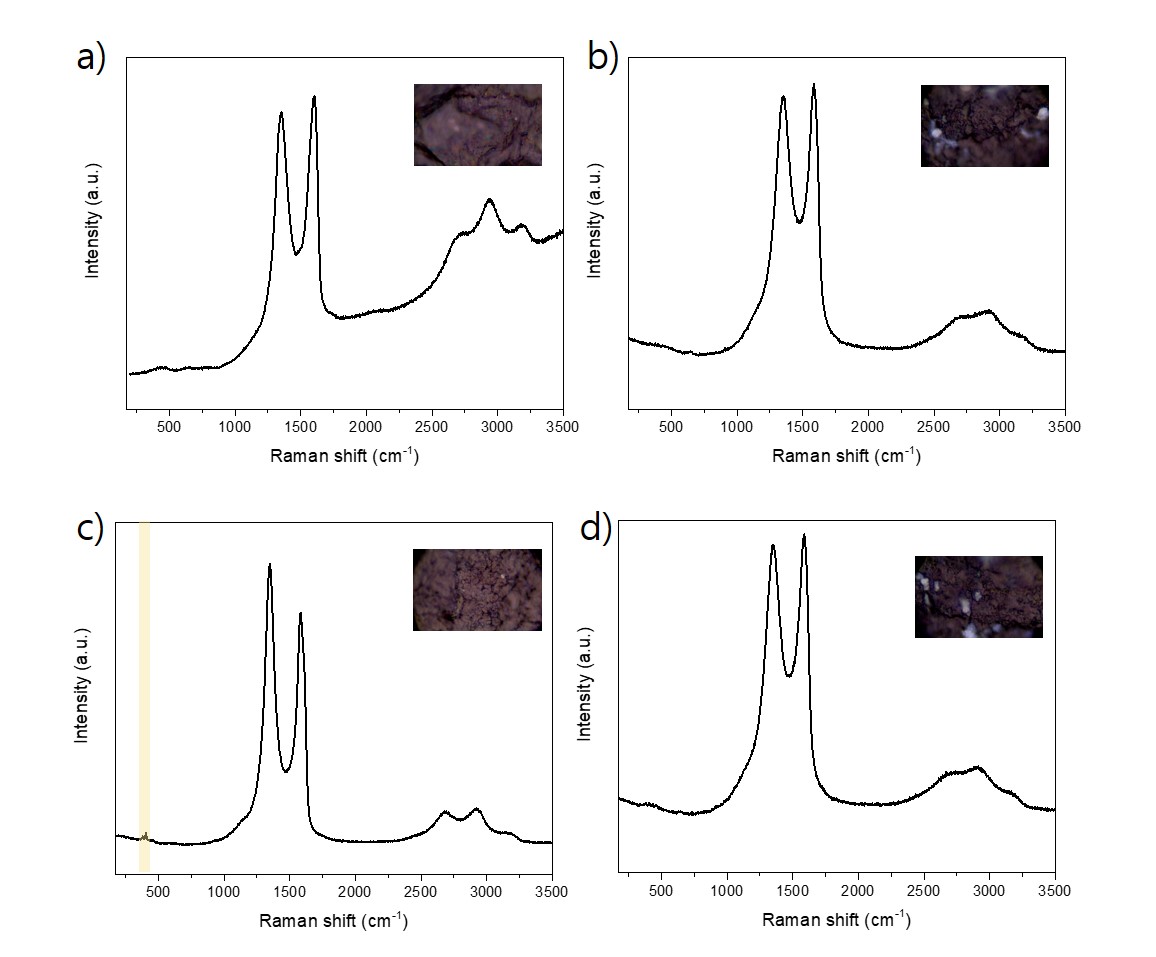


Figure S1. Raman spectrum of rGO/1T-MoS_2_ prepared by vacuum filtration. (a) and (b) show the film with a volume ratio of rGO to MoS_2_ = 7:1. The Raman spectrum vary depending on the specific location where the measurement is conducted. (c) and (d) are the film with a volume ratio of rGO to MoS_2_ = 7:5.

**Figure S2**. (a-f) Energy-dispersive X-ray spectroscopy (EDS) element mapping obtained from SEM images, depicting the distribution of carbon (C), oxygen (O), molybdenum (Mo), and sulfur (S) in rGO/1TMoS_2_ composite. (g-I) EDS elemental mapping acquired from TEM analysis.


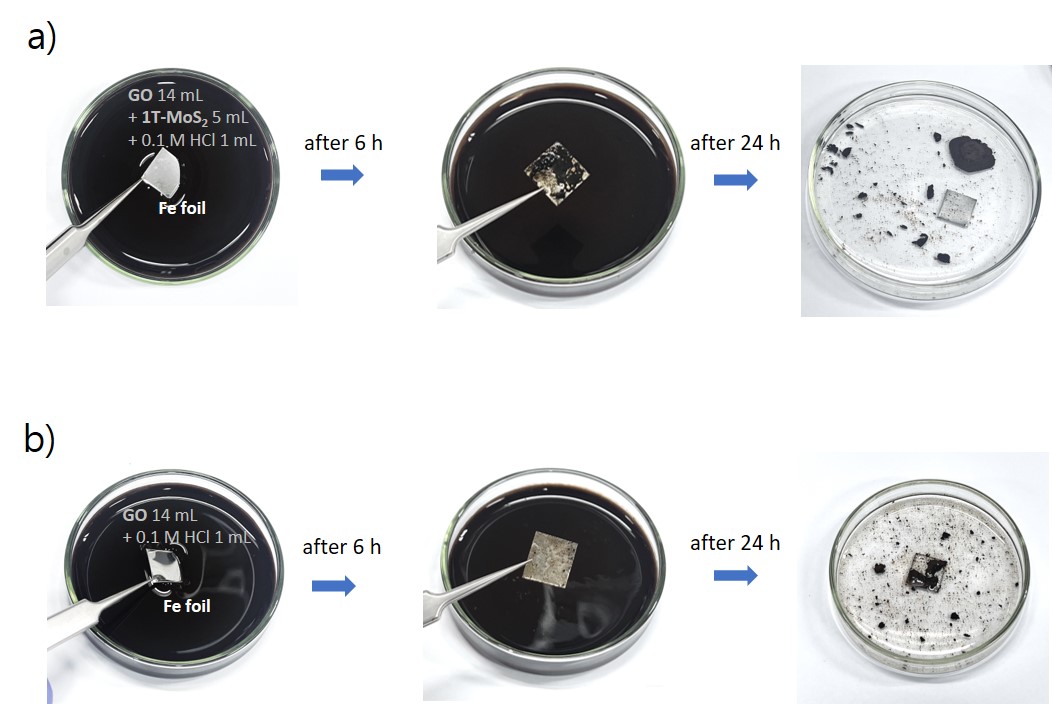


Figure S3. Reduction of GO on Fe foil. (a) The formation of the film occurs faster when MoS_2_ is added. (b) The rGO film without MoS_2_.


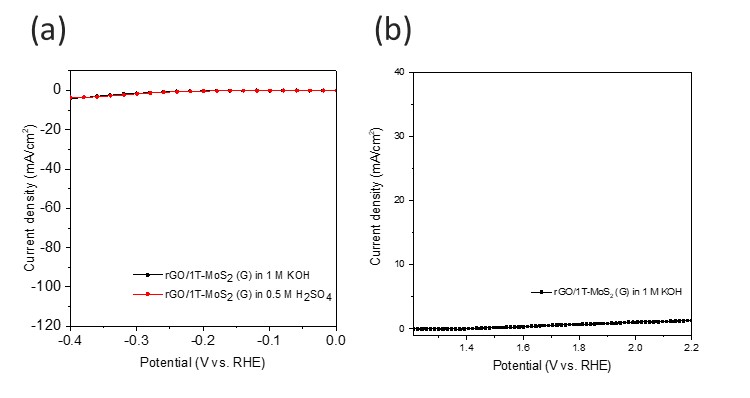


**Figure S4**. Polarization curves of rGO/1T-MoS_2_ (G) film as substrates. (a) HER in both acid and alkaline electrolytes and OER (b) in alkaline electrolyte.


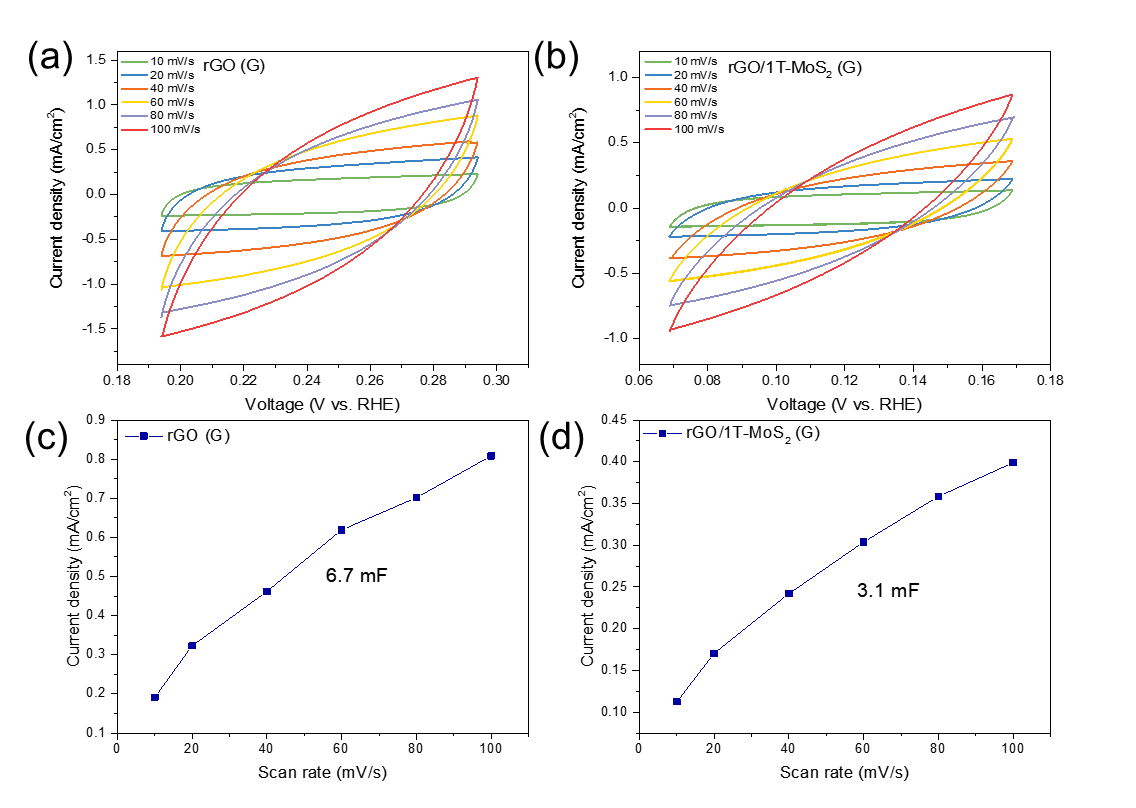


**Figure S5**. Measurement of electrochemically active surface area (ECSA). (a) rGO (G) film, (b) rGO/1T-MoS_2_ film (G). (c and d) Double layer capacitance of rGO (G) film and rGO/1T-MoS_2_ (G) film. The ECSA of rGO and rGO/1T-MoS_2_ film is determined through the measurement of the double-layer capacitance (Cdl) which shows 6.7 mF/cm^2^ and 3.1 mF/cm^2^,respectively. Consequently, the roughness factors of rGO and rGO/MoS_2_ are determined to be 11.96 and 8.67, respectively. The geometrical area of the electrode of rGO and rGO/MoS_2_ is 0.56 cm^2^ and 0.36 cm^2^, respectively. Therefore, ECSA of rGO and rGO/MoS_2_ is calculated to be 6.7 cm^2^ and 3.1 cm^2^, respectively.

Table 1. Electrical conductivity of rGO/MoS_2_ composites

| Sample | Fabrication Method | Conductivity (S/cm) | Reference |
| --- | --- | --- | --- |
| MoS_2_/Graphene Composite Paper  (MoS_2_:rGO = 1:5) | Vacuum membrane filtration, followed by further reduction at elevated temperatures. | 1.86 | [1] |
| MoS_2_/Graphene Composite Paper  (MoS_2_:rGO = 2:5) | Vacuum membrane filtration, followed by further reduction at elevated temperatures. | 1.17 | [1] |
| MoS_2_/Graphene Composite Paper  (MoS_2_:rGO = 3:5) | Vacuum membrane filtration, followed by further reduction at elevated temperatures. | 0.47 | [1] |
| MoS_2_/Graphene Nanosheet (GNS)  (MoS_2_:GNS = 9:1) | Liquid-phase ultrasound method | 2.693 | [2] |
| MoS_2_/Graphene Nanosheet (GNS)  (MoS_2_:GNS = 8:2) | Liquid-phase ultrasound method | 3.545 | [2] |
| MoS_2_/Graphene Nanosheet (GNS)  (MoS_2_:GNS = 7:3) | Liquid-phase ultrasound method | 1.420 | [2] |
| rGO fiber with 20 wt% MoS_2_ doping | Wet-spinning method | 144 | [3] |
| rGO fiber with 40 wt% MoS_2_ doping | Wet-spinning method | 59 | [3] |
| MoS_2_-rGO nanocomposite | Refluxing method | 0.19 at 323 K | [4] |
| MoS_2_-graphene aerogel | Thermal decomposition method | 1.12 | [5] |
| MoS_2_/Graphene Nanosheet (GN) hybrids  (10 wt% of GN) | Liquid phase stripping, Hydrothermal method | 1.095 | [6] |
| rGO-MoS_2_-0.25 | Hydrothermal method | 0.6553 | [7] |
| rGO-MoS_2_-0.30 | Hydrothermal method | 0.9166 | [7] |
| rGO-MoS_2_-0.33 | Hydrothermal method | 1.133 | [7] |
| rGO-MoS_2_-0.40 | Hydrothermal method | 3.334 | [7] |
| MoS_2_/rGO | Hydrothermal method | 0.435 | [8] |
| NCMTs@A-MoS_2_/RGO film  (N doped carbon microtubes@amorphous MoS_2_/RGO) | Pressed pellet | 0.0098 | [9] |
| MoS_2_/C nanocomposite | Hydrothermal method | 0.0238 | [10] |
| 0.5 MoS_x_/r-GO | One-pot solution process/Freezed dried gel | 0.057 | [11] |
| 0.7 MoS_x_/r-GO | One-pot solution process/Freezed dried gel | 0.032 | [11] |
| CC/MoS2@RGO-700 | Cotton textile-derived carbon/MoS_2_@RGO (By encapsulating a thin film of RGO | 0.42 | [12] |

**References**

1. L. David, R. Bhandavat, and G. Singh ACS Nano 2014, 8, 2, 1759
2. J.H. Choi, M.-C Kim, S.-H. Moon, H. Kim, Y.-S. Kim, and K.-W. Park RSC Adv., 2020, 10, 19077
3. J. Li and H. Wang et al J. Mater. Chem. A, 2019, 7, 3143
4. A. K. Gautam, M. Faraz, and N. Khare Journal of Alloys and Compounds*,* 2020, 838, 155673
5. M. A. Worsley, S. J. Shin, M. D. Merill, J. Lenhardt, A. J. Nelson, L. Y. Woo, A. E. Gash T. F. Baumann, and C. A. Orme ACS Nano*,* 2015, 9, 5, 4698-4705
6. J. Chai, D. Zhang, J. Cheng, Y. Jia, X. Ba, Y. Gao, L. Zhu, H. Wang, and M. Cao RSC Adv., 2018, 8, 36616
7. X. Ding, Y. Huang, S. Li, N. Zhang, J. Wang Composites Part A: Applied Science and Manufacturing 2016, 90, 424
8. R. Illathvalappil, S. M. Unni, and S. Kurungot Nanoscale, 2015, 7, 16729
9. X. Liu, X. Zhang, S. Ma, S. Tong, X. Han, and H. Wang Electochinica Acta, 2020, 135568
10. X. Lit, X. Zhang, S. Ma, S. Tong, X. Han, and H. Wang Journal of Alloys and Compounds, 2017, 729, 583
11. C.-H. Lee, J.-M. Yun, S. Lee, S. M. Jo, K. Eom, D. Lee, H.-I Joh, and T. F. Fuller Scientific reports, 2017, 7, 41190
12. X. Liu, H. Ji, B. Peng, Z. Cui, Q. Liu, Q. Zhao, L. Yang, and D. Wang Inor. Chem. Fornt. 2023, 10, 267
